# Histone modifications induced by MDV infection at early cytolytic and latency phases

**DOI:** 10.1186/s12864-015-1492-6

**Published:** 2015-04-18

**Authors:** Apratim Mitra, Juan Luo, Yanghua He, Yulan Gu, Huanmin Zhang, Keji Zhao, Kairong Cui, Jiuzhou Song

**Affiliations:** Department of Animal & Avian Sciences, University of Maryland, College Park, MD 20742 USA; USDA, ARS, Avian Disease and Oncology Laboratory, East Lansing, MI 48823 USA; Systems Biology Center, National Heart, Lung and Blood Institute, National Institutes of Health, Bethesda, MD 20892 USA; Department of Animal Breeding and Genetics, College of Animal Sciences, China Agricultural University, Beijing, 100193 P.R. China

## Abstract

**Background:**

Marek’s disease (MD) is a highly contagious, lymphomatous disease of chickens induced by a herpesvirus, Marek’s disease virus (MDV) that is the cause of major annual losses to the poultry industry. MD pathogenesis involves multiple stages including an early cytolytic phase and latency, and transitions between these stages are governed by several host and environmental factors. The success of vaccination strategies has led to the increased virulence of MDV and selective breeding of naturally resistant chickens is seen as a viable alternative. While multiple gene expression studies have been performed in resistant and susceptible populations, little is known about the epigenetic effects of infection.

**Results:**

In this study, we investigated temporal chromatin signatures induced by MDV by analyzing early cytolytic and latent phases of infection in the bursa of Fabricius of MD-resistant and –susceptible birds. Major global variations in chromatin marks were observed at different stages of MD in the two lines. Differential H3K27me3 marks were associated with immune-related pathways, such as MAP kinase signaling, focal adhesion and neuroactive ligand receptor interaction, and suggested varying degrees of silencing in response to infection. Immune-related microRNAs, e.g. *gga-miR-155* and *gga-miR-10b*, bore chromatin signatures, which suggested their contribution to MD-susceptibility. Finally, several members of the focal adhesion pathway, e.g. *THBS4* and *ITGA1*, showed marked concordance between gene expression and chromatin marks indicating putative epigenetic regulation in response to MDV infection.

**Conclusion:**

Our comprehensive analysis of chromatin signatures, therefore, revealed further clues about the epigenetic effects of MDV infection although further studies are necessary to elucidate the functional implications of the observed variations in histone modifications.

**Electronic supplementary material:**

The online version of this article (doi:10.1186/s12864-015-1492-6) contains supplementary material, which is available to authorized users.

## Background

Marek’s disease (MD) is a highly infectious disease caused by an α-herpesvirus, Marek’s disease virus (MDV) that affects chickens worldwide. MD pathogenesis can be divided into three major stages: an early cytolytic phase, which occurs between 3 and 6 days post infection (dpi), is characterized by the infection of B lymphocytes, the first major targets of MDV. The infected B cells enter circulation and induce the activation of CD4+ T cells which in turn become infected. Subsequently, CD4+ T cells form the primary vehicle for MDV multiplication and dissemination, along with a smaller percentage of other cells including B and CD8+ T lymphocytes. Around 7 dpi, the infection enters a latent phase defined by the absence of expressed viral antigens and virus production. This switch to latency is believed to be governed by many viral and host factors, such as, viral interleukin (vIL)-8, which acts as a chemoattractant for T lymphocytes [1], and upregulated chicken major histocompatibility complex (MHC) class II molecules on infected cells promoting the initiation of host immune response [2]. In MD-resistant chickens, latent infection persists at a low level in lymphoid tissues and CD4+ T lymphocytes. However, in MD-susceptible genotypes, a second cytolytic phase occurs 2–3 weeks after the primary infection, wherein latently infected lymphocytes are transformed and proliferate rapidly to form tumors in various tissues.

The primary lymphoid organs of spleen, thymus and the bursa of Fabricius are important focal points of MD progression. Cytolytic infection initiates in the spleen before spreading to other lymphoid organs, which lag behind by a day. This is accompanied by significant cytolysis of B and T lymphocytes in addition to varying levels of inflammatory response. Bursal follicles and the thymic cortex undergo regressive changes in this stage of MD leading to organ weight loss, while there is massive apoptosis of thymocytes. In the spleen, however, inflammation results in an increase in organ weight. The above changes are reduced within two weeks of infection, with the organs almost returning to their normal form and structure. However, in MD-susceptible chickens, a second wave of cytolytic infection around 14-21dpi results in marked inflammation, severe atrophy of bursa and thymus and permanent immunosuppression.

Our prior studies of host response to latent MDV in spleen and thymus [3,4], revealed associations between histone modifications and immune-related functions. In spleen, MDV-challenged chickens showed changes in epigenetic status of genes related to cell adhesion, G-protein coupled signaling pathways and ion transport. Also, the expression of various miRNAs appeared to be regulated by the trimethylation of lysine 4 and 27 of histone H3. Further, widespread differential histone modification enrichments were detected in the thymus at genes related to cancer and host response to viruses. We also detected bivalent domains on immune-related transcriptional regulators in both MD-susceptible and resistant chicken lines. Thus, it was clear that MDV infection induced significant epigenetic changes in the birds, and the individual response was dependent on genetic background.

The bursa of Fabricius is the primary site for lymphocyte B-cell development and antibody repertoire formation in birds. There have been several studies of the effect of MD, particularly in the spleen, but relatively few concerning the bursa of Fabricius [5,6]. The latter is a primary lymphoid organ evolutionarily unique to birds and critical to the development of the B cell lineage [7]. B lymphocytes in all the major lymphoid organs are the primary targets of the virus in the early stages of the disease [8]. Embryonal bursectomy resulted in the abolition of early lytic infection along with reduced viremia and tumor formation, in spite of comparable MD incidence [6]. Bursal atrophy was observed in MD-susceptible line L7_2_ chickens with the effect reduced in the MD-resistant line L6_3_ individuals [8], while the bursa-dependent immune system was impaired in infected chickens [9]. It is, therefore, evident that the bursa of Fabricius plays an important role in MD pathogenesis, and it is vitally important to understand its immunological effect of MDV infection.

To address the above issues, we performed a temporal analysis of chromatin signatures induced by MDV infection in inbred chicken lines having contrasting responses to the disease. The tissue of interest was the bursa of Fabricius and we included both the cytolytic and latent phases of MD in this study. The biological consequences of chromatin profiles are context-specific and similar patterns can lead to a variety of outcomes [10]. Therefore, we focused on *changes* of chromatin enrichments as evidence of possible epigenetic regulation. Our primary goal was to associate the dynamic changes of chromatin induced by MDV infection to the underlying biological pathways to reveal the functional effects of the viral infection. Due to the inherent complexities of such experiments, it is difficult to separate cause from effect, but the results could provide further clues about this complex disease and define future avenues of research.

## Results

### Genome-wide histone modification profiling

We sampled two critical time-points of MD progression, 5 and 10 dpi, representing early cytolytic and latent stages of MD, respectively. Chromatin immunoprecipitation followed by massively parallel sequencing (ChIP-Seq) was performed on bursal tissues obtained from MD-resistant line L6_3_ and MD-susceptible line L7_2_ chickens. Two histone H3 trimethylation marks having opposing effects on gene regulation were profiled – H3 lysine 4 trimethylation (H3K4me3), which is associated with the 5’ end of active genes, and H3 lysine 27 trimethylation (H3K27me3), which marks broad regions for silencing. More than 390 million reads from 32 samples were mapped to the chicken genome and analyzed using the WaveSeq peak-calling algorithm [4]. Subsequently, peaks were merged to obtain unambiguous regions of enrichment and annotated with overlapping genes.

To verify data quality, we calculated the correlation between biological replicates using read counts in a 2 kilobase region around the transcription start sites of annotated genes (−1000 bp to +1000 bp). Pearson correlation coefficients between replicates were excellent, with a majority above 95% suggesting highly consistent ChIP-Seq experiments (Additional file 1). The median number of peaks detected was 15126 for H3K4me3 and 46850 for H3K27me3 (Additional file 2). The number of enrichment regions and associated genes were comparable across time points (Table 1), with a majority of peaks for both histone marks present in all conditions. These observations were consistent with our earlier studies [3,4], which showed that these histone marks occupied largely similar genomic regions in both lines, irrespective of infection status. Thus, the changes induced by MDV infection likely involved subtle variations at specific genomic loci rather than *de novo* establishment or abrogation of histone marks.

**Table 1:** **Histone modification peaks and associated genes by dpi**

	**Histone**	**DPI**	**Merged Peaks**	**Filtered**	**Gene Overlaps**	**Unique Genes**
By DPI	H3K4me3	5	18100	15115	6430	7499
		10	29625	16855	6488	7530
	H3K27me3	5	94461	51290	24520	11776
		10	98105	56294	27057	12035
Across DPI	H3K4me3			17161	6645	7730
	H3K27me3			47602	24339	13044

Merged peaks are filtered by removing those appearing in only one sample. ‘Gene Overlaps’ refers to number of peaks that overlapped annotated genes.

### Differential chromatin marks indicate functional differences in MD response

We obtained a set of unambiguous enrichment regions at each dpi by merging peak calls across samples. Subsequently, read counts were calculated within these regions and comparisons between samples carried out using edgeR [11] with a false discovery rate of 0.1 used to define differentially marked regions (DMRs). We used four pair-wise comparisons in our analysis – two of these were ‘within-line’ comparisons, wherein infected and control birds from lines L6_3_ and L7_2_ were compared amongst themselves. The remaining two were ‘between-line’ comparisons, in which either control or infected birds from the two lines were compared (L63v72N and L63v72I, respectively). Differences detected in within-line comparisons arose primarily due to MDV infection, while variations detected in the L63v72I comparison would represent disparities in disease response. On the other hand, the L63v72N comparison would reveal baseline differences between the two lines.

### DMRs induced by MDV infection

The within-line comparisons yielded striking differences between the two lines. The susceptible line L7_2_ displayed large numbers of differential H3K4me3 and H3K27me3 marks at 5 dpi, while a similar phenomenon was observed in line L6_3_ at 10 dpi (Table 2).To examine functional significance, the MD-induced DMRs were annotated with overlapping genes and analyzed for functional enrichments using DAVID [12,13].

**Table 2:** **DMRs detected in within- and between-line comparisons**

**Within-line comparisons**							
		**L6**_**3**_ **infected vs control**			**L7**_**2**_ **infected vs control**		
**Histone Mark**	**DPI**	**DMRs**	**Mapped to Genes**	**Unique Genes**	**DMRs**	**Mapped to Genes**	**Unique Genes**
H3K4me3	5	53	46	52	1820	1241	1478
	10	13458	7744	8184	72	41	51
H3K27me3	5	0	0	0	8775	3489	2330
	10	3865	2056	2573	12	9	11
**Between-line comparisons**							
		**L6**_**3**_ **vs L7**_**2**_ **control**			**L6**_**3**_ **vs L7**_**2**_ **infected**		
**Histone Mark**	**DPI**	**DMRs**	**Mapped to Genes**	**Unique Genes**	**DMRs**	**Mapped to Genes**	**Unique Genes**
H3K4me3	5	2071	1253	1369	6427	4269	4639
	10	498	259	271	2613	1651	2088
H3K27me3	5	3454	1394	707	13807	5881	4355
	10	2550	1557	2136	2937	1599	1725

DPI: days post infection; DMR: Differentially marked region; Mapped to genes: Number of DMRs that overlap genes; Unique genes: Number of unique genes overlapped.

Several interesting KEGG pathways were significantly enriched among the genes displaying MD-induced DMRs (Table 3).The ubiquitin-mediated proteolysis pathway, which is associated with various processes such as cell cycle and inflammatory response [14,15] showed significant variations in H3K4me3 marks at the cytolytic stage of infection in the susceptible line and also at the latent stage of infection in the resistant line. Similarly, variations in H3K27me3 levels were observed in both lines but at different time-points of infection, on genes linked to the cell proliferation-associated mitogen-activated protein (MAP) kinase signaling [16] and Wnt signaling pathways [17]. H3K27me3 DMRs were also observed on genes associated with the focal adhesion pathway, which affects cancer cell migration [18] and the neuroactive ligand receptor-interaction pathway, a collection of signaling molecules and receptors. Interestingly, the cell cycle pathway, the dysregulation of which is a hallmark of cancer, and the spliceosome pathway which regulates alternative splicing, displayed H3K4me3 DMRs unique to line L6_3_ at 10 dpi.

**Table 3:** **Selected KEGG pathways enriched in MD-induced DMRs**

**Histone**	**DPI**	**Line**	**KEGG pathway**	**Count**	**P-value**	**Adjusted p-value**	**FDR**
H3K4me3	5	L7_2_	gga04120:Ubiquitin mediated proteolysis	20	0.011523	0.258461	12.60988
	10	L6_3_	gga03040:Spliceosome	76	1.27E-07	1.83E-05	1.5x10^−4^
			gga04120:Ubiquitin mediated proteolysis	66	0.070518	0.409344	57.98318
			gga04110:Cell cycle	61	0.071502	0.39873	58.50744
H3K27me3	5	L7_2_	gga04512:ECM-receptor interaction	21	5.79E-04	0.070325	0.667972
			gga04080:Neuroactive ligand-receptor interaction	45	0.004334	0.239385	4.905434
			gga04510:Focal adhesion	32	0.0364	0.540977	34.90982
			gga04010:MAPK signaling pathway	37	0.052973	0.533259	46.75692
			gga04310:Wnt signaling pathway	23	0.097339	0.6588	69.45459
	10	L6_3_	gga04080:Neuroactive ligand-receptor interaction	48	0.001017	0.12119	1.172739
			gga04010:MAPK signaling pathway	38	0.041861	0.595512	39.09871
			gga04510:Focal adhesion	32	0.042927	0.548881	39.87981
			gga04310:Wnt signaling pathway	24	0.069217	0.679767	56.47578

DPI: Days post infection; Count: Number of genes associated with pathway; FDR: False discovery rate. P-value, adjusted p-value and FDR were calculated using default parameters in DAVID [12,13].

Various immune-related gene ontology (GO) terms were also enriched among genes associated with MD-induced DMRs, suggesting differing outcomes of MD in the two lines (Additional file 3).For instance, at 5 dpi in line L7_2_,enriched GO categories were associated with stress and DNA damage response (H3K4me3), cell-cell signaling, biological adhesion and cell proliferation (H3K27me3). A similar scenario was observed in line L6_3_ at 10 dpi, in addition to terms associated with regulation of apoptosis, lymphocyte activation and cytokine production. Thus, subtle differences in enriched functional terms hinted at possible variations in underlying immune response. Overall, the two lines displayed similar effects of MDV infection, but at different points of disease progression suggesting a ‘phase-difference’ in epigenetic effects of MDV infection.

### Epigenetic differences between resistant and susceptible lines

On comparing the two lines, several differences were observed between the control birds, but a greater number of DMRs were obtained from the L63v72I comparison (Table 2). At 5 dpi among control birds, genes associated with immune-related GO terms, e.g. regulation of cytokine production, various signaling pathways, regulation of apoptosis and cell proliferation, displayed perturbed H3K4me3 enrichments, while terms related to signaling pathways showed variations in H3K27me3 levels. This phenomenon was also observed at the latent stage of infection, but with a few notable differences, e.g. T cell activation was enriched among H3K27me3 DMRs. These results suggested baseline epigenetic differences between the two lines, which could contribute to innate differences in immune response.

Symptomatic of the highly divergent responses to MDV infection in the two lines, infected birds exhibited more than twice as many DMRs as the control birds over the two time-points. Functional analysis revealed a wide array of enriched functional terms and pathways. Notable examples included MAP kinase signaling, Wnt signaling, focal adhesion and neuroactive ligand-receptor interaction pathways, which were also enriched in H3K27me3 DMRs from within-line comparisons. In addition, larger numbers of immune-related GO terms were enriched in H3K4me3 and H3K27me3 DMRs at 5 dpi, such as, cell cycle, cell proliferation and apoptosis.

Thus, comparisons of the MD-resistant and susceptible lines revealed innate differences in histone modification profiles in addition to variations induced by MDV infection. Several immune-related pathways and GO functional terms were enriched among genes displaying differential chromatin marks, indicating the functional significance of such epigenetic differences.

### Critical pathways exhibit common H3K27me3 signatures

**Figure 1:**
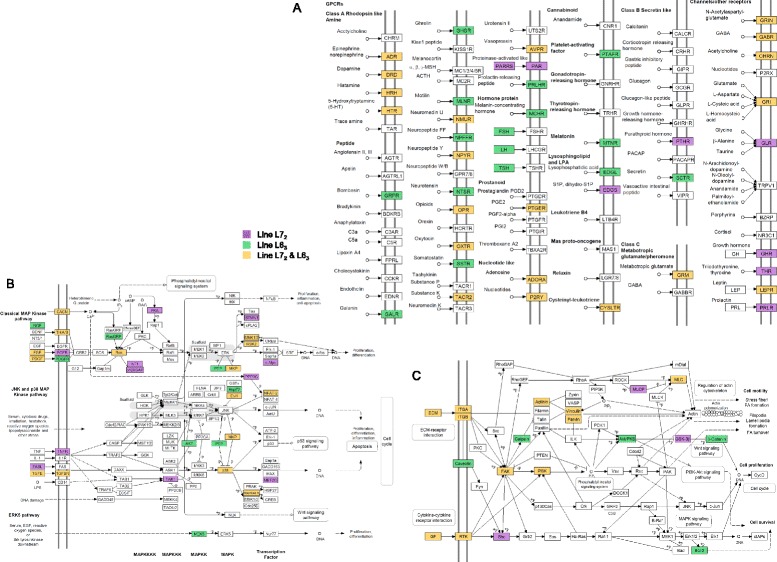
Critical KEGG pathways exhibit common H3K27me3 signatures. **A)** MAP kinase signaling; **B)** Neuroactive ligand-receptor interaction; **C)** Focal adhesion signaling. Several genes from these pathways display a common H3K27me3 pattern: there are increased repressive marks at 5 dpi in the susceptible line and at 10 dpi in the resistant line, while overall levels are higher in the resistant line. Genes showing the common chromatin signature are represented by filled boxes.

Strong statistical enrichment and association with multiple comparisons or time-points can be considered to be indicative of functional relevance and several of the above enriched pathways satisfied this criterion (Figure 1). Interestingly, the neuroactive-ligand receptor interaction pathway and immune-related pathways such as, MAP kinase signaling (Figure 1A) and focal adhesion were associated with H3K27me3 DMRs in both lines and shared a common chromatin signature. A majority of genes exhibited increased H3K27me3 levels after infection in the susceptible line at 5 dpi and in the resistant line at 10 dpi, suggesting epigenetic silencing at different stages of MD. However, these pathways also displayed higher H3K27me3 levels in infected birds from line L6_3_ compared to line L7_2_, indicating higher levels of silencing in the resistant line in response to infection. To further investigate the functional significance of these variations, we examined the underlying genes associated with DMRs in the above pathways.

Neuroactive ligand-receptor interaction (Figure 1B), as mentioned above, is a collection of signaling molecules, such as, hormones and neurotransmitters, and their corresponding receptors. Several classes of G protein-coupled receptors, e.g. dopamine, 5-hydroxytryptamine (5-HT) and histamine receptors, in addition to a variety of others, such as, leptin, glutamate and γ-aminobutyric acid (GABA) receptors, displayed H3K27me3 variations in both lines (Figure 1B). However, there were some important differences as well. The cytotoxic T-lymphocyte and natural killer cell-specific serine protease, granzyme A (*GZMA*), and the growth hormone receptor (*GHR*), both displayed increased H3K27me3 levels in infected L7_2_ birds at 5 dpi. *GZMA* was upregulated in spleen tissues of MDV-infected chickens irrespective of genetic background [19], *GHR* was upregulated in resistant birds at 10 dpi [20]. In bursa tissues, *GZMA* was upregulated in both lines at 10 dpi, but no difference was detected in *GHR* transcript levels. Certain DMRs were unique to the resistant line, e.g. the platelet-activating factor receptor *PTAFR*, which exhibited higher H3K27me3 marks in infected birds from line L6_3_.

MAP kinase signaling pathways regulate a wide variety of cellular processes ranging from cell proliferation to apoptosis [21] and have been linked to MD-induced tumorigenesis via the virus-encoded oncogene Meq [22]. In the classical MAP kinase pathway, growth factors, e.g. fibroblast growth factors (FGFs), activate Ras-related proteins to trigger protein kinase cascades involving Raf, MEK and ERK, leading to varying outcomes including cell proliferation and cell differentiation. Moreover, extracellular signals, such as, reactive oxygen species and stress, can activate the p38 and JNK MAP kinase pathways via cytokines, e.g. TGFβ and IL-1, leading to proliferation and apoptosis. Several *FGF* and *RAS* genes, in addition to multiple calcium channel genes, displayed H3K27me3 DMRs in both lines, suggesting potential hot-spots of epigenetic regulation. H3K27me3 DMRs were also observed on various members of the p38 and JNK MAP kinase pathways, such as, p38 MAP kinases, *MAPK12* and *MAPK13*, and immune-related genes, e.g. *TGFB2*, *NFATC2*.

Focal adhesions consist of macromolecular protein complexes that connect the actin cytoskeleton of a cell to the extra-cellular matrix (ECM) (Figure 1C). Focal adhesions are also sites of integrin-mediated signal transduction which plays critical roles in cell migration and angiogenesis [23]. Several genes encoding extra-cellular matrix proteins, e.g. various collagens, laminins and integrins, in addition to actinin and vinculin displayed H3K27me3 changes in both lines. Moreover, several growth factors, e.g. *PDGFA*, and receptor tyrosine kinases, were also associated with H3K27me3 DMRs and were possible sites of epigenetic regulation.

Thus, important immune-related pathways, e.g. MAP kinase signaling and focal adhesion, exhibited H3K27me3 DMRs which suggested potential hot-spots of epigenetic silencing and regulation. Moreover, immune-related genes belonging to the neuroactive ligand-receptor interaction pathway displayed H3K27me3 variations unique to each line indicating the presence of differing epigenetic responses to MDV infection.

### Immune-related microRNAs are associated with differential chromatin marks

Various classes of non-coding transcripts have been subjects of intense study in recent years, and as a result, regulatory roles for many such species, e.g. microRNAs (miRNAs), have been uncovered. MiRNAs are a class of small non-coding RNAs that regulate gene expression at the post-transcriptional level. Several regulatory roles for miRNAs have been discovered, ranging from immune response, inflammation and tumorigenesis [24-27]. Thus, in addition to annotated protein-coding genes, we were interested in investigating non-coding transcripts associated with differential histone marks. We downloaded microRNA annotations from miRBase [28] and searched for nearby DMRs and genes to reveal functionally important miRNAs (Additional file 4).

Based on their position relative to nearby genes, miRNAs can be classified into three categories: intergenic, intragenic (sense orientation) and intragenic-reverse (anti-sense orientation). We found H3K27me3 and H3K4me3 DMRs associated with 198 unique miRNAs, a majority of which (109 out of 198; 55%) were intergenic. The small size of miRNAs relative to DMRs leads to two issues; first, it is difficult to attribute chromatin marks to miRNAs that overlap larger protein-coding genes. Second, the overall fold-difference for a large DMR could be different in the vicinity of the miRNA, leading to false positives. Thus, to account for the above, we concentrated on intergenic miRNAs for subsequent analysis.

**Figure 2:**
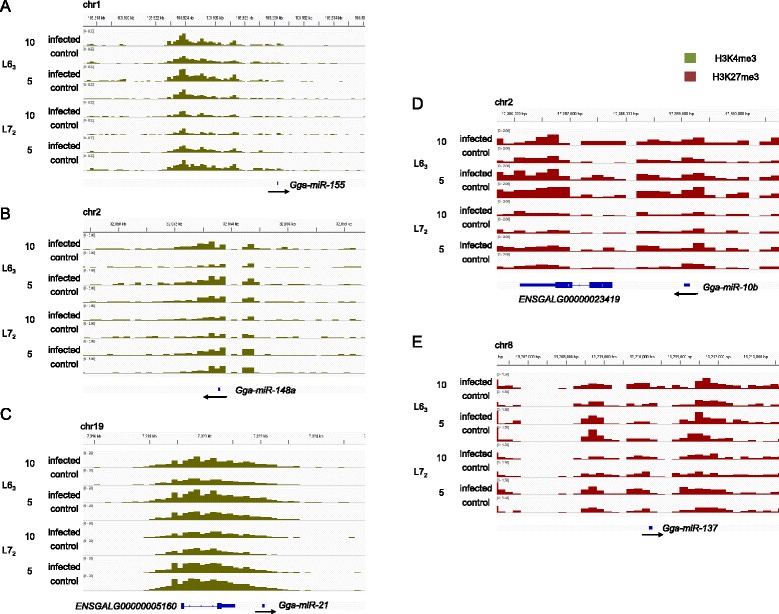
MicroRNAs with immune-related functions are associated with differential chromatin marks. **A)** *Gga-mir-155*, **B)** *Gga-mir-148a* and **C)** *Gga-mir-21* display significantly increased H3K4me3 marks in the resistant line L6_3_ at the latent stage of infection. There are no observed differences between the lines at the cytolytic stage although levels of H3K4me3 appear to be higher. **D)** *Gga-mir-10b* and **E)** *Gga-mir-137* exhibit increased H3K27me3 marks in line L6_3_ at 10 dpi, while both infected and control L7_2_ birds have high H3K27me3 levels. Both lines show low levels of H3K27me3 at the earlier stage of infection.

Close examination of the list of DMR-associated miRNAs revealed several immune-related miRNAs, e.g. *gga-miR-155*, *gga-miR-148a* (H3K4me3), *gga-miR-10b* and *gga-miR-137* (H3K27me3). The widely studied miRNA, miR-155, is critical for normal B cell differentiation [29] and plays a major role in immune response and inflammation by regulating members of the tumor-necrosis factor superfamily [30]. MiR-155 also controls antiviral CD8+ T cell responses by regulating interferons, and mir-155-deficient mice had reduced viral clearance [31]. All samples exhibited strong H3K4me3 marks around *gga-miR-155* at both time-points, suggesting activation, but the chromatin marks were higher in the resistant line L6_3_ particularly at 10 dpi (Figure 2A). MiR-148a induces apoptosis in colorectal cancer [32] and increases cell proliferation in gastric cancer [33]. *Gga-miR-148a* displayed increased H3K4me3 marks in infected birds from the resistant line at 10 dpi, while no changes were evident at the earlier time-point (Figure 2B). The first discovered mammalian miRNA, miR-21, has been implicated in a wide variety of cancers as it targets tumor suppressors for repression [34-36] (Figure 2C). Similar to *gga-miR-155*, strong H3K4me3 marks were observed at the promoter of *gga-miR-21* in all samples, with a slight reduction in line L7_2_ at 10 dpi (Figure 2A).

MiR-10b is highly upregulated in breast cancer and triggers metastasis of tumor cells [37]. At 5 dpi, line L6_3_ displayed high levels of H3K27me3 around*gga-miR-10b* both before and after infection, compared to L7_2_ birds (Figure 2D). Meanwhile, at 10 dpi, only infected birds from the resistant line L6_3_ showed high H3K27me3 levels at this locus. MiR-137 acts as a tumor suppressor in neuroblastoma by downregulating a histone demethylase [38] and also regulates cell migration and proliferation in breast cancer [39]. MDV-infected line L7_2_ birds displayed increased levels of H3K27me3 compared to control birds, on *gga-miR-137* at 5 dpi, while line L6_3_ displayed higher levels irrespective of infection status (Figure 2E). At the latter time-point, H3K27me3 marks were reduced in control birds from both lines, but line L6_3_ displayed increased H3K27me3 levels in infected birds.

Thus, several miRNAs with immune-related functions showed changes in histone modifications and were likely subject to epigenetic regulation as a result of MDV infection. The varying responses in the two chicken lines particularly around *gga-miR-155*, *gga-miR-10b* and *gga-miR-137*, also suggested the possible contribution of these miRNAs to differential MD-resistance*.*

### Differential chromatin and differential gene expression

Having investigated global chromatin profiles, we were interested in examining the correlation between chromatin marks and gene expression levels. Our prior studies [3,4] had revealed mixed results – absolute expression levels correlated well with H3K4me3 (positive) and H3K27me3 (negative) levels, but no relationship was apparent between differential expression and differential histone marks. Similarly, in the current study, we found moderate correlation between chromatin marks and absolute gene expression (Additional file 5), but low overlap between differential chromatin and differential transcript levels. However, in spite of modest global correlations, transcriptional changes consistent with epigenetic variations for certain loci could indicate epigenetic regulation. Thus, to find genes whose expression levels correlated with chromatin marks, we carried out a systematic comparison of DMR-associated genes and differentially expressed genes from RNA-Seq experiments carried out in the same tissue. Subsequently, we focused on functionally important DMRs to discover possible candidates for further analysis.

**Figure 3:**
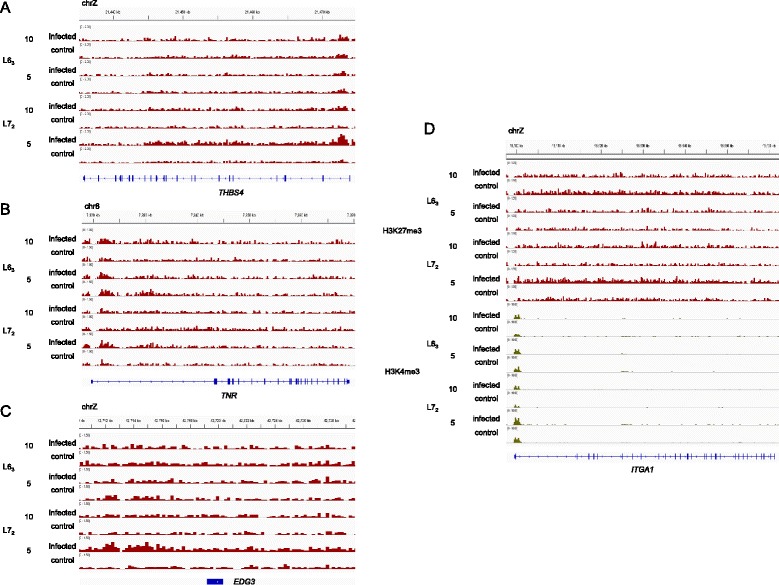
Differential H3K27me3 marks associated with differential gene expression. **A)** *THBS4*, **B)** *TNR*, and **C)** *EDG3* genes showed increased repressive H3K27me3 marks in susceptible line L7_2_ as a result of cytolytic MD infection that is accompanied by significant downregulation. **D)** *ITGA1*displayed reduced H3K27me3 enrichments and significant upregulation in the resistant line at 10 dpi as a result of MD infection. The susceptible line displayed increased H3K27me3 marks in line L7_2_ at 5 dpi, but there was no accompanying change in gene expression.

Relatively few differentially expressed genes were associated with DMRs enriched in the pathways outlined above, with H3K27me3 DMRs at 5 dpi being a notable exception. Thrombospondin 4 (*THBS4*), a member of the focal adhesion pathway, is an adhesive glycoprotein that plays a major role in proliferation and development of erythroid cell lineages, while also mediating cell-cell signaling and cell-matrix interactions [40]. Recently, this gene was found to be downregulated in various gastric tumors [41] and shown to have tumor suppressor properties [42]. *THBS4* displayed significantly increased H3K27me3 levels (Figure 3A) and was also highly downregulated (fold-change = −4.58x, FDR = 3.68x10^−4^) in MD-infected susceptible chickens at 5 dpi. Tenascin-R (TNR), belongs to a family of extra-cellular matrix proteins, which are involved in regulating cell adhesion [43]. Other members of the tenascin family, TNC and TNW, are associated with disease, with increased expression correlating with higher cell motility and loss of focal adhesions, while TNR performs multiple functions in the central nervous system [44]. Higher H3K27me3 levels around *TNR* (Figure 3B), accompanied reduced transcript levels, in MD-infected line L7_2_ birds at 5 dpi. Endothelial differentiation sphingolipid-G receptor 3 (EDG3), is a G protein-coupled receptor that contributes to the regulation of cell migration [45] and vascular endothelial cell function [46]. EDG3 is necessary for the stimulation of the serine threonine kinase, Akt3, by the vascular endothelial growth factor (VEGF), and reduced levels of Akt kinase leads to increased apoptosis of cancer cells [47]. *EDG3* displayed increased H3K27me3 levels in line L7_2_ birds in response to MDV infection at 5 dpi (Figure 3C), which was coupled with significantly lower transcript levels (fold-change = −1.8x, FDR = 0.0879).

One of the few genes that was differentially expressed and associated with a strong H3K27me3 DMR in line L6_3_ was *integrin alpha 1* (*ITGA1*). *ITGA1* encodes the alpha 1 subunit of integrin receptors for collagen and laminin on the cell surface, which regulate cell-cell adhesion and may be involved in inflammation. *ITGA1* displayed reduced H3K27me3 levels (Figure 3D) and increased expression after infection in resistant birds at 10 dpi (fold-change = 3.16x, FDR = 0.0757). Interestingly, line L7_2_ birds exhibited higher H3K27me3 and H3K4me3 marks in response to infection at 5 dpi, but no change in gene expression was observed.

Thus, there was limited overlap between differential chromatin marks and differentially expressed genes, with the majority attributable to H3K27me3 DMRs observed at 5 dpi in the susceptible line. Integrated analysis of ChIP-Seq and RNA-Seq experiments revealed interesting candidates for further analysis, e.g. *THBS4* and *ITGA1*. However, the low concordance between the two independent experiments highlights the complexity of the chromatin landscape and the multitude of regulatory factors involved in determining transcription and phenotype.

## Discussion

The chromatin code is a universal, multi-layered guide to the transcriptional regulatory machinery that allows tremendous diversity to be encoded into the genome, while providing an essential link between the genetic material and environmental cues. Interpreting the biological consequences of variations in chromatin marks is exceedingly complex and can be likened to an attempt to discern the outcome of a voluminous treatise from its preface. The task of understanding the broader genomic effects of a complex disease, such as MD, from epigenetic profiling is a similarly daunting undertaking. Our prior studies of the epigenetic effects of latent MD on resistant and susceptible chicken lines [3,4] have provided us with some perspective. Here, we expanded the scope of such studies by investigating two critical stages of MD progression.

There were striking global differences in chromatin modifications between the two lines, as we observed a large number of MD-induced DMRs in line L7_2_ at 5 dpi and in line L6_3_ at 10 dpi. Important immune-related pathways, e.g. MAP kinase signaling and focal adhesion, were associated with H3K27me3 DMRs in both lines, indicating functional similarities in epigenetic response at different stages of MDV infection. However, the same pathways displayed higher H3K27me3 levels in infected line L6_3_ birds compared to line L7_2_ suggesting a greater degree of silencing in the resistant line. The MAP kinase signaling pathway has been widely associated with proliferation [16] and is targeted by the Meq protein to induce viral transformation [22]. Our results suggest that this pathway might be preferentially silenced in the resistant line by H3K27me3, which leads to increased transformation and tumorigenesis in susceptible birds.Several G protein-coupled receptors, members of the neuroactive ligand-receptor interaction pathway, were also associated with H3K27me3 DMRs in both lines. However, certain differences were also apparent, notably *GZMA* and *GHR*, which showed increased H3K27me3 in L7_2_ birds at 5 dpi in response to MDV infection. The functional significance of these chromatin changes is unclear as there was no apparent effect on gene expression. In the resistant line, H3K4me3 DMRs were associated with the cell cycle and spliceosome pathways, but observed fold-changes were small and therefore, they were not chosen for further analysis.

In addition to protein-coding genes and pathways, several cancer-related miRNAs were associated with both H3K4me3 and H3K27me3 DMRs. Lower H3K4me3 levels and possible reduction in transcript levels around *gga-miR-155* in line L7_2_could contribute to higher MD-susceptibility. Two miRNAs associated with seemingly opposite effects in breast cancer, *gga-miR-10b* and *gga-mir-137*, both exhibited putative epigenetic silencing in the resistant line. MiR-10b initiates tumor invasion and metastasis in breast cancer [37], while miR-137 reduces the proliferative and migratory capacities of breast cancer cells [39]. Apart from *Gga-mir-155* [48] none of the above miRNAs have been previously reported in the context of MD and their roles in determination of MD-resistance bear further analysis. Also, we focused our attentions on intergenic miRNAs in this study to avoid the ambiguities involved in overlapping genes and miRNAs. However, it is equally important to investigate the roles of other classes of miRNAs in the context of MD-resistance and susceptibility and will be the subject of future work.

The global comparison of histone modifications and gene expression revealed moderate correlations, while the overlap between differentially expressed genes and differential chromatin enrichments was low. As mentioned above, this could be a consequence of the complexity of transcriptional regulatory mechanisms and relatively few factors assayed. However, our data can be used to infer the location of hotspots of epigenetic regulation. Using this approach we identified several genes, a majority of which were associated with H3K27me3 DMRs and whose expression correlated well with observed chromatin marks. Three members of the focal adhesion pathway, *THBS4*, *TNR* and *ITGA1*, displayed increased H3K27me3 levels in infected line L7_2_ birds at 5 dpi. In the case of *THBS4* and *TNR*, this was accompanied by a significant downregulation, while *ITGA1* displayed a concordant increase in H3K4me3 marks but no change in gene expression. On the other hand, in line L6_3_, infected birds showed a reduction of H3K27me3 levels around *ITGA1*at 10 dpi and a corresponding upregulation, while there were no significant changes in chromatin marks or gene expression on *THBS4* or *TNR. THBS4* is a putative tumor suppressor [42] and its downregulation could be associated with increased MD-susceptibility in line L7_2_ birds. Moreover, the dysregulation of ECM genes and receptors is a hallmark of cancer metastasis. Our data suggests that this pathway is a hotspot of epigenetic regulation as a consequence of MDV infection and merits further investigation in the context of MD-resistance.

## Conclusions

In summary, we conducted a comprehensive analysis of the chromatin landscape induced by MDV in two inbred chicken lines with contrasting responses to the disease. We found major global variations in chromatin marks occurring at different stages of infection in the two lines. Functional analysis of genes associated with differential H3K27me3 enrichments revealed enriched pathways, such as, MAP kinase signaling, focal adhesion and neuroactive ligand-receptor interaction that were shared between lines. However, infected birds from lines L6_3_ and L7_2_ displayed different H3K27me3 levels on members of these pathways, indicating varying degrees of silencing in response to infection. In addition, several immune-related miRNAs, e.g. *gga-miR-155* and *gga-miR-10b*, were associated with differential chromatin marks and could contribute to increased MD-susceptibility in line L7_2_ chickens. Finally, several members of the focal adhesion pathway, *THBS4*, *TNR* and *ITGA1*, were associated with differential chromatin and transcript levels, indicating that this pathway may by a hotspot of epigenetic regulation in response to MDV infection. Taken together, our results shed further light on the epigenetic effects of MD, revealing striking global differences and possible functional impact of epigenetic variations. Additional functional assays are necessary to elucidate the underlying mechanisms behind the large-scale chromatin changes and the effect on MD-resistance and susceptibility.

## Methods

### Animals and viruses

Two specific-pathogen-free inbred lines of White Leghorn, either resistant (L6_3_) or susceptible (L7_2_) to MD, were hatched, reared and maintained in Avian Disease and Oncology Laboratory (ADOL, Michigan, USDA). Eight chickens from each line were injected intra-abdominally with a partially attenuated very virulent plus strain of MDV (648A passage 40) at 14 days after hatch with a viral dosage of 500 plaque-forming units (PFU). Another eight chickens were not infected as age-matched controls. Infected and control chickens (n = 4) from both lines were terminated at 5 or 10dpi to collect bursa tissues. All procedures followed the standard animal ethics and use guidelines of ADOL (31320-008-00D) and University of Maryland (R-08-62).

### Analysis of ChIP-Seq data

Chromatin immunoprecipitation (ChIP) was carried out using bursa samples from MDV infected and control birds as described elsewhere [8]. Briefly, about 30 mg bursa samples were digested with micrococcal nuclease followed by end-repair with PNK and Klenow enzymes (NBE, Ipswich, MA, USA) and ChIP with the specific antibody. This was followed by addition of 3’ adenine, Illumina adaptor ligation, PCR amplification (17 cycles) and size-selection (~150 bp), cluster generation and sequencing on the Illumina Hi-Seq 2000. Sequence reads were aligned to the May 2006 version of the chicken genome (galGal3) using bowtie version 0.12.7 [49]. Default alignment policies of bowtie were enforced: a valid alignment could have a maximum of two mismatches and if a read aligned equally well to multiple places in the genome, one was chosen at random. If multiple reads mapped to the same genomic location, only one was kept to avoid amplification bias. The total number of reads obtained for each sample is listed in Additional file 6. For visualization purposes, read counts were combined from two replicates of each sample and normalized to reads per million mapped reads (RPM). Between-replicate correlations were calculated using normalized reads in a 2 kb region around the TSS, with low-scoring regions in both replicates (bottom 25%) not included in the calculation. We also plotted the density of the normalized reads around the TSS averaged over two replicates of each sample (Additional file 7). The distributions of both H3K4me3 and H3K27me3 were uniform across all samples with no outliers.

Peak-calling was carried out using the WaveSeq algorithm [4]. We used recommended values for parameters: for H3K4me3 data, the mother function was ‘morlet’ and gap size was 2 (400 bp), while for H3K27me3, the mother function was ‘mexican hat’ and gap size was 10. The p-value threshold for H3K27me3 was also lowered to 0.4. Peaks detected in the same genomic region of multiple samples were merged to include all peaks and those appearing in only one sample were removed as possible false positives. Filtered peaks were annotated with genes based on overlaps in the transcription start site (TSS) region for H3K4me3 and gene body (TSS to transcription termination site, TTS) for H3K27me3. Gene annotations consisted of 16426 genes from the Ensembl database Release 66 [50], which included a majority of RefSeq genes in addition to predicted genes and miRNAs.

### Functional analysis of DMRs

Reads mapping to filtered peaks were tabulated into a matrix and analyzed using edgeR [11] and statistical significance defined using a false discovery rate of 0.1.Functional analysis of DMRs was performed with DAVID [12,13] using default parameters. Highly enriched KEGG pathways were selected based on degree of statistical significance (FDR < 0.3) and biological relevance. If a pathway was significantly enriched for a particular comparison, other instances of this pathway were retained for closer examination.

### MicroRNAs associated with DMRs

MiRNA annotations were downloaded from miRBase [28] version 18 and annotated with nearby DMRs and genes. MiRNAs with complete or partial overlaps with genes were classified as intragenic or intragenic-reverse, based on relative orientation. The maximum distance for annotation with a DMR was chosen to be 1 kb.

### Availability of supporting data

The data discussed in this publication have been deposited in NCBI’s Gene Expression Omnibus [51] and are accessible through GEO Series accession number GSE65961 (http://www.ncbi.nlm.nih.gov/geo/query/acc.cgi?acc=GSE65961).

## Additional files

Additional file 1:
**Correlation between replicates.** High correlation observed between replicates of samples in present study. Correlation coefficients correspond to Pearson correlation between normalized read counts in a 2 kb region flanking the TSS (−1000 bp to +1000 bp). Additional file 2:
**Peak-calling results.** The number of peaks called in each sample using the WaveSeq algorithm. Additional file 3:
**Selected GO terms enriched in MD-induced DMRs.** Enrichment analysis results from DAVID. Additional file 4:
**MiRNAs associated with DMRs.** Locations of significant peaks overlapping microRNAs are shown, in addition to any protein-coding genes that are overlapped. Additional file 5: Figures S1-S4. ChIP-Seq vs RNA-Seq – S1) L63, 5dpi, S2) L72, 5dpi, S3) L63, 10dpi, S4) L72, 10dpi. Moderate to low correlation observed between ChIP-Seq and RNA-Seq experiments with H3K4me3 marks positively correlated and H3K27me3 negatively correlated with gene expression. Genes were divided into groups of 100 based on absolute expression. ChIP-Seq read counts were calculated in the promoter region (TSS ± 500 bp) for H3K4me3 and in the gene body (TSS to TTS) for H3K27me3.The ChIP-Seq read counts and RNA-Seq read counts wereaveraged for genes within each group and plotted on scatterplots (open black circles). The linear best fit line was drawn (red) and the corresponding Pearson correlation coefficients are shown in individual plots. Additional file 6:
**Sequencing statistics.** The numbers of reads obtained from sequencing the samples used in this study before are shown. Additional file 7:
**Distribution of mean RPKM around TSS.** The densities of mean RPKM from a 2 kb region surrounding the TSS for a) H3K27me3 and b) H3K4me3 data. All samples showed uniform distributions for both histone marks.
