# Supplementary material for: Histone modifications induced by MDV infection at early cytolytic and latency phases
Source: BMC Genomics. 2015 Apr 18;16(1):311. doi: 10.1186/s12864-015-1492-6 (PMC4404578; doi:10.1186/s12864-015-1492-6)

Figure S1. ChIP-Seq vs RNA-Seq – $L6_3$, 5dpi

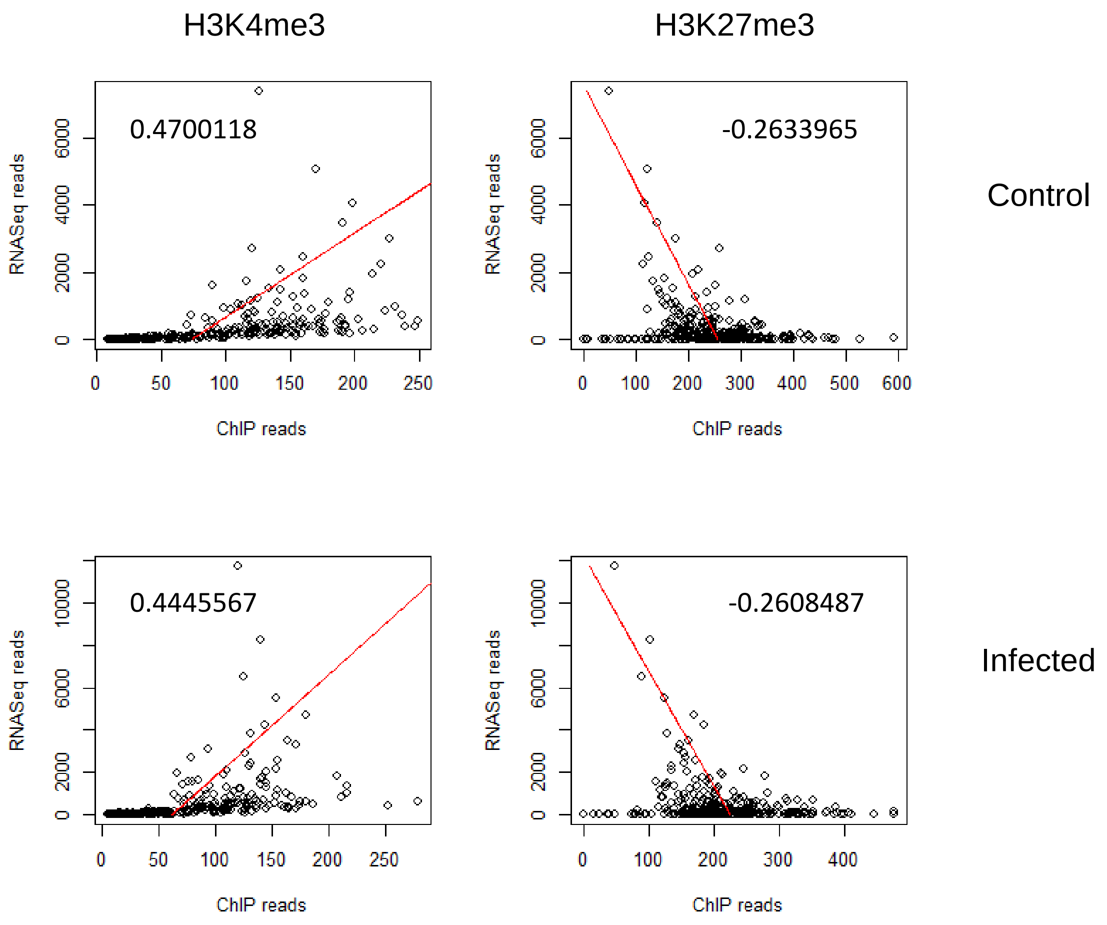

Figure S2. ChIP-Seq vs RNA-Seq – $L7_2$, 5dpi

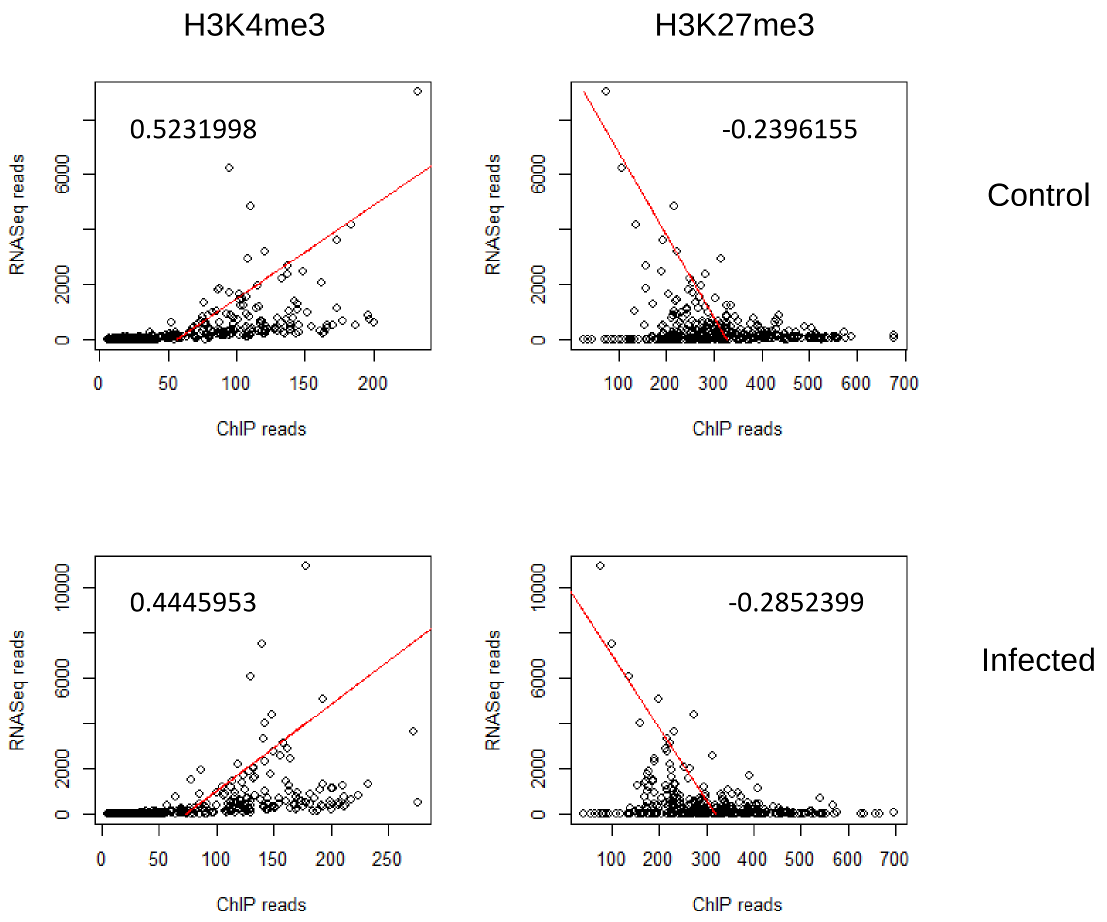

Figure S3. ChIP-Seq vs RNA-Seq – $L6_3$, 10dpi

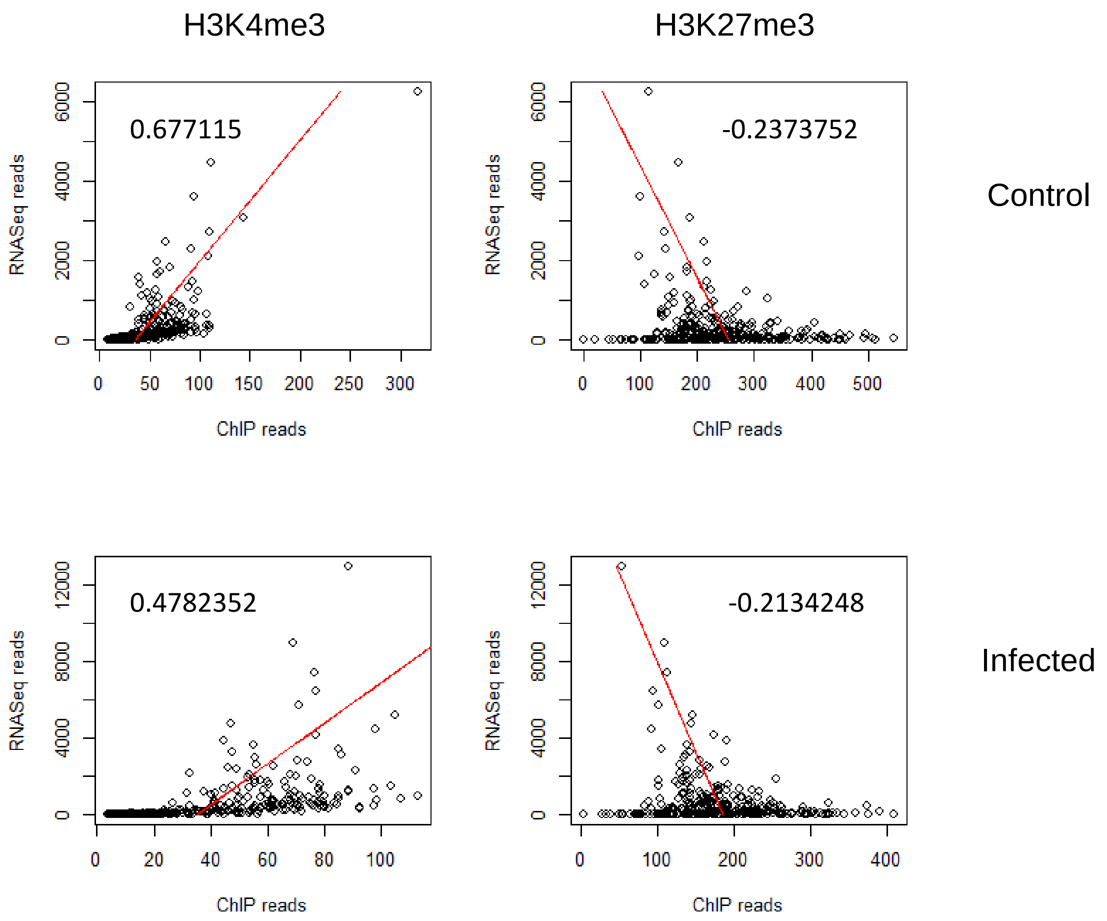

Figure S4. ChIP-Seq vs RNA-Seq – $L7_2$, 10dpi

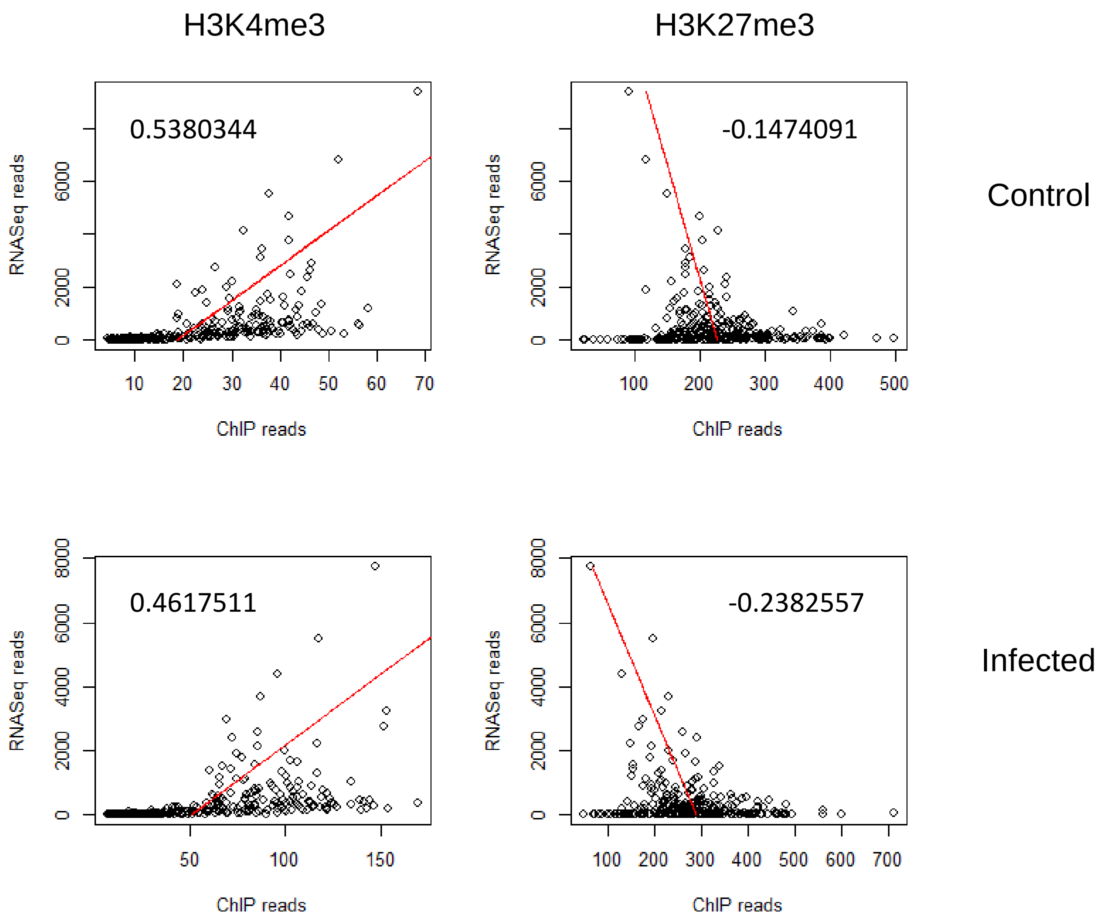

Supplement: Additional file 5: Figures S1-S4. — ChIP-Seq vs RNA-Seq – S1) L63, 5dpi, S2) L72, 5dpi, S3) L63, 10dpi, S4) L72, 10dpi. Moderate to low correlation observed between ChIP-Seq and RNA-Seq experiments with H3K4me3 marks positively correlated and H3K27me3 negatively correlated with gene expression. Genes were divided into groups of 100 based on absolute expression. ChIP-Seq read counts were calculated in the promoter region (TSS ± 500 bp) for H3K4me3 and in the gene body (TSS to TTS) for H3K27me3.The ChIP-Seq read counts and RNA-Seq read counts wereaveraged for genes within each group and plotted on scatterplots (open black circles). The linear best fit line was drawn (red) and the corresponding Pearson correlation coefficients are shown in individual plots. [file 12864_2015_1492_MOESM5_ESM.pdf]
