# Supplementary figures and images for: Histone modifications induced by MDV infection at early cytolytic and latency phases
Source: BMC Genomics. 2015 Apr 18;16(1):311. doi: 10.1186/s12864-015-1492-6 (PMC4404578; doi:10.1186/s12864-015-1492-6)

### H3K27me3

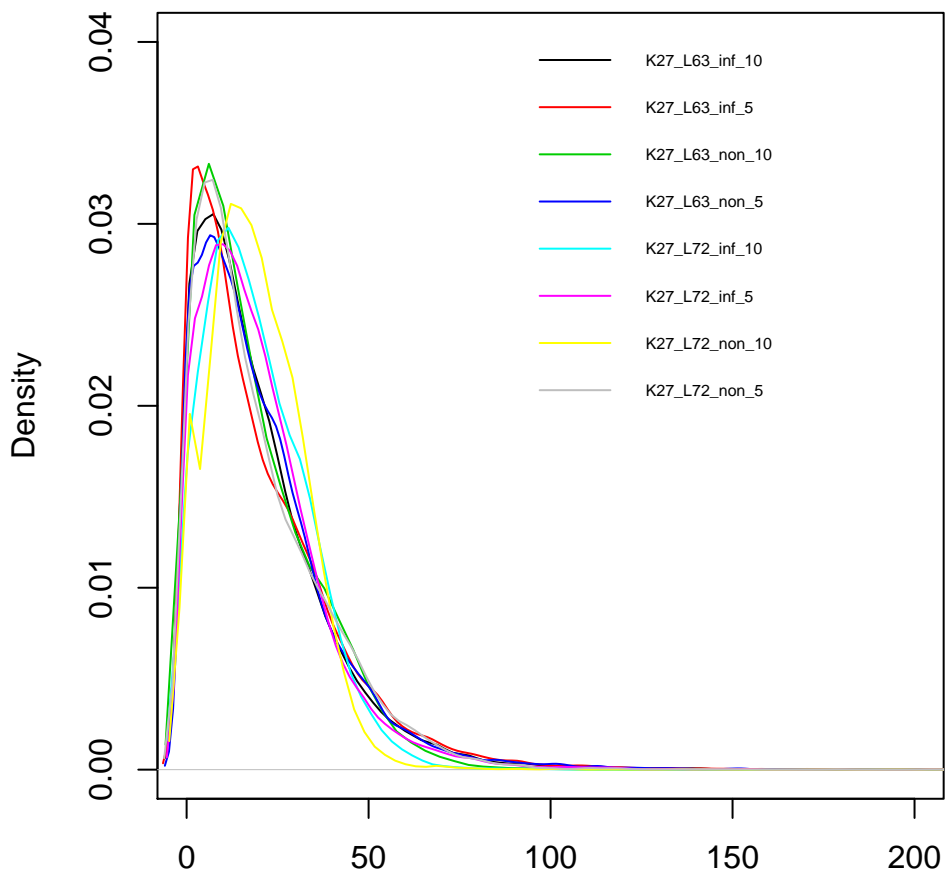

N = 23389 Bandwidth = 1.942

### H3K4me3

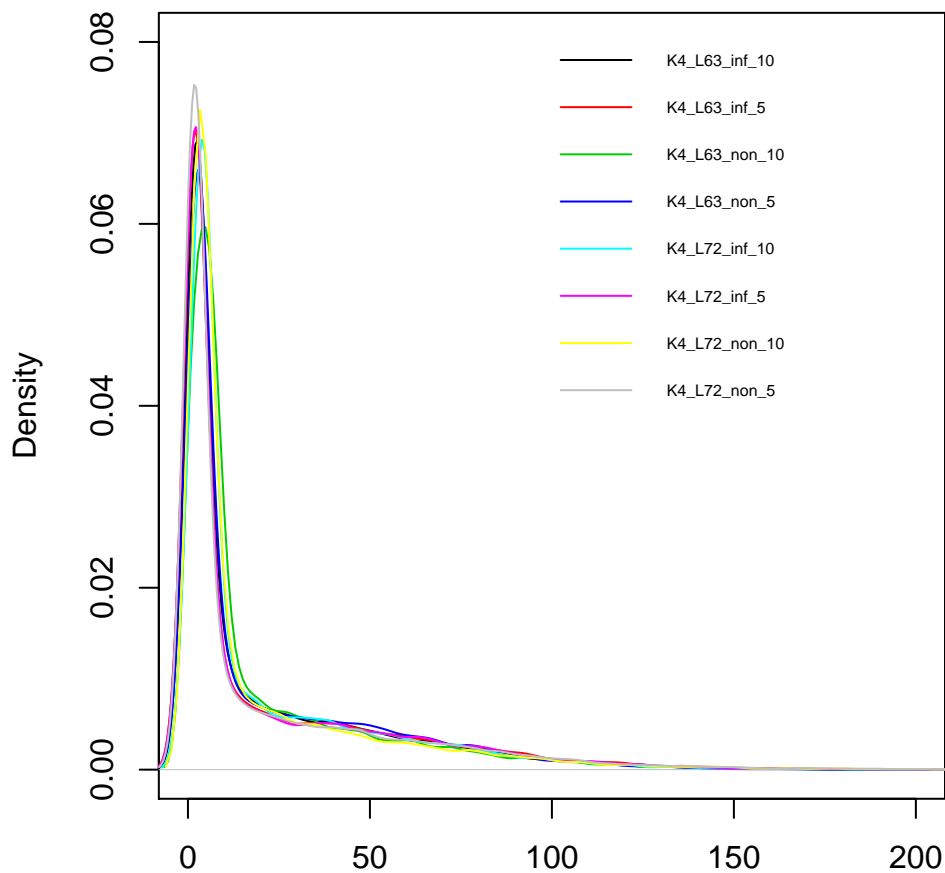

N = 23389 Bandwidth = 2.62

Supplement: Additional file 7: — Distribution of mean RPKM around TSS. The densities of mean RPKM from a 2 kb region surrounding the TSS for a) H3K27me3 and b) H3K4me3 data. All samples showed uniform distributions for both histone marks. [file 12864_2015_1492_MOESM7_ESM.pdf]
